# Altered phenotype and gene transcription in endothelial cells, induced by *Plasmodium falciparum*-infected red blood cells: Pathogenic or protective?

**DOI:** 10.1016/j.ijpara.2007.02.006

**Published:** 2007-07

**Authors:** Srabasti J. Chakravorty, Celine Carret, Gerard B. Nash, Al Ivens, Tadge Szestak, Alister G. Craig

**Affiliations:** a Molecular & Biochemical Parasitology, Liverpool School of Tropical Medicine, University of Liverpool, Liverpool, L3 5QA, United Kingdom; b The Wellcome Trust Sanger Institute, Hinxton, Cambridge, CB10 1SA, United Kingdom; c Department of Physiology, University of Birmingham, Birmingham, B15 2TT, United Kingdom

**Keywords:** Severe malaria, Vascular endothelium, Co-culture, ICAM-1, Microarray, Tumour necrosis factor

## Abstract

Severe malaria is associated with sequestration of *Plasmodium falciparum*-infected red blood cells (PRBC) in the microvasculature and elevation of intercellular adhesion molecule-1 (ICAM-1) and TNF. In vitro co-culture of human umbilical vein endothelial cells (HUVEC), with either PRBC or uninfected RBC, required the presence of low level TNF (5 pg/ml) for significant up-regulation of ICAM-1, which may contribute to increased cytoadhesion in vivo. These effects were independent of *P. falciparum* erythrocyte membrane protein-1 (PfEMP-1)-mediated adhesion but critically dependent on cell–cell contact. Further changes included increases in IL8 release and soluble TNF receptor shedding. Microarray analysis of HUVEC transcriptome following co-culture, using a human Affymetrix microarray chip, showed significant differential regulation of genes which defined gene ontologies such as cell communication, cell adhesion, signal transduction and immune response. Our data demonstrate that endothelial cells have the ability to mobilise immune and pro-adhesive responses when exposed to both PRBC and TNF. In addition, there is also a previously un-described positive regulation by RBC and TNF and a concurrent negative regulation of a range of genes involved in inflammation and cell-death, by PRBC and TNF. We propose that the balance between positive and negative regulation demonstrated in our study will determine endothelial pathology during a malaria infection.

## 1 Introduction

Pathogenesis of severe malaria has stirred considerable interest over the years, however there are many questions at the mechanistic level that remain unanswered. Severe malaria is characterised, in part, by sequestration of *Plasmodium falciparum*-infected red blood cells (PRBC) at microvascular sites in the brain tissue, leading ultimately to coma and subsequent death. There is evidence that the sequestration of PRBC on the endothelial surface can result in loss of integrity, thus compromising blood–brain barrier function either directly (Brown et al., 1999a,b, 2001a), by indirect mechanisms including binding via platelets (Wassmer et al., 2004; Combes et al., 2006) or by the production of microparticles from endothelial cells (EC) activated by adhering PRBC (Combes et al., 2005, 2006), and also cytokine-driven modulation of endothelial cell metabolism, but that this is usually a small, transient effect in vivo.

One question is the mechanism underpinning the accumulation of PRBC leading to micro-vascular occlusion as seen in post mortem cerebral malaria (CM) brain tissue, which is believed to be a progressive phenomenon of PRBC sequestration. We suggest that the initial sequestration of PRBC, if maintained for a prolonged period of time, has the ability to activate the endothelium to promote sequestration, leading to deleterious effects on the host. Our studies investigate the direct effects on the endothelium of PRBC retention for prolonged periods of time.

First, sustained exposure of the endothelium to PRBC can result in modulation of the endothelium, thus increasing its responsiveness to low levels of TNF (TNF^low^). This may contribute to promoting further cytoadhesion in microvasculature containing sequestered PRBC. This hypothesis is based on previous evidence of endothelial cell activation by abnormal red blood cells in various conditions, including sickle cell disease, diabetes and also malaria (Shiu and McIntire, 2003). Sickle cell RBC (sRBC), diabetic RBC (dRBC) and also PRBCs can all induce expression of adhesion molecules such as intercellular adhesion molecule-1 (ICAM-1) and vascular adhesion molecule-1 (VCAM-1) and E-selectin (Brown et al., 2001b; Shiu and McIntire, 2003; Viebig et al., 2005; Tripathi et al., 2006). In addition, recent co-culture studies have demonstrated PRBC-induced increases in the activity of pro-apoptotic genes involved in the caspase pathway (Pino et al., 2003). Other changes induced on EC by sRBCs include induction of endothelin and prostacyclin production (Shiu et al., 2002) and by PRBC include increased expression of pro-inflammatory genes (Pino et al., 2003) and release of IL6 (Viebig et al., 2005).

Serum levels of IL8 were elevated in severe malaria compared with healthy volunteers (Lyke et al., 2004), correlated with parasite count and severity of disease at the time of admission (Burgmann et al., 1995) and there were no apparent differences between febrile and a-febrile volunteers (Hermsen et al., 2003). IL8 is an endothelial-derived acute response activation marker. Prestored IL8 is exocytosed at a basal level from Wiebel palade bodies in ECs and is rapidly released in response to various stimuli (Wolff et al., 1998; Utgaard et al., 1998; Oynebraten et al., 2004).

Exposure of ECs to sRBCs in vitro resulted in increased sensitivity to the pro-inflammatory cytokine IL1β (Shiu et al., 2000). We propose that local increases in TNF levels occur in the microvasculature in early stages of infection, during shizogony (Bisser et al., 2006). In a similar fashion to that observed with sRBCs, contact between PRBCs and the endothelium could potentially sensitise the ECs to TNF. There is evidence for an important role for TNF receptors (TNFR) in CM pathology, however, most of this comes from studies on animal models. Lucas et al. (1997a) showed a close correlation between absence of TNFR II expression and protection from CM-associated brain damage in TNFR II knock-out mice, while TNFR II was up-regulated on brain endothelium in CM-susceptible mice (Lucas et al., 1997b). Up-regulation of the soluble TNFR I, which is the predominant type in human serum, was reported in the serum of patients with acute *P. falciparum* infection (Wenisch et al., 1994). Interestingly, EC from CM-susceptible mice had a greater sensitivity to TNF than that of CM-resistant mice. TNF induced up-regulation of TNFR I and II together with an associated increase in IL6, ICAM-1 and VCAM-1 to a greater extent in CM-susceptible mice than in CM-resistant mice (Lou et al., 1998). In addition, there was an absence of ICAM-1 up-regulation in TNFR II knock-out mice, suggesting a link between TNFRs and ICAM-1 up-regulation during malaria infection (Lucas et al., 1997a).

Our aim was to investigate the ability of PRBC to modulate the endothelium in the presence and absence of the inflammatory cytokine, TNF, in a co-culture system. Firstly, functional markers of EC modulation included endothelial expression of ICAM-1, which has been attributed a critical role in parasite adhesion, and the release of IL8. Changes in levels of TNFR I and II, were also investigated as a potential mechanism for any changes in the sensitivity of EC to TNF. Second, we sought to assess the global transcriptional changes in ECs and elucidate the regulation of cellular processes following co-culture under the same conditions, using a human genome Affymetrix (Affymetrix, Santa Clara, CA, USA; http://www.affymetrix.com) chip.

Our results have led us to propose a novel mechanism for the modulation of the endothelium during malaria infection that is dependent on low level TNF and involves a pro-inflammatory component but also a concurrent down-modulation of RBC-induced inflammation due to the presence of the parasite within the infected cell.

## 2 Materials and methods

### 2.1 Malarial parasites

*Plasmodium falciparum* ItG strain was derived from the Brazilian line IT4/25/5 (Ockenhouse et al., 1992). This strain was used for the PRBCs in these studies. The ItG strain is a strong ICAM-1 binder and also binds to CD36 (Gray et al., 2003). The PRBCs were cultured in RPMI-1640 supplemented with 2 mM l-glutamine, 37.5 mM *N*-2-hydroxyehtylpiperazine-*N*′-2-ethanesulfonic acid (Hepes), 10 mM glucose, 25 μg/ml gentamicin and 10% human serum, at pH of 7.2 (Trager and Jensen, 1976). All reagents were obtained from Sigma, UK. Human serum was isolated from whole blood obtained from the Royal Liverpool Hospital and was approved by the Liverpool School of Tropical Medicine Ethical Committee.

### 2.2 Endothelial cells

Pooled human umbilical vein endothelial cells (HUVEC) were obtained from Promocell (Heidelberg, Germany), HUVEC from different batches were used for each experiment, at passages three to five. HUVEC were grown to confluence on 1% gelatin (Sigma, UK) coated flasks and plates. All co-culture experiments were performed in serum-depleted basal HUVEC medium (quiescing medium) which consisted of M199 (Invitrogen, UK) containing 1% FCS. These conditions were designed to increase the signal window, while maintaining the integrity of the HUVEC monolayer (i.e. there was no indication of cell apoptosis or necrosis during the experiments).

For all co-culture studies, ECs were co-cultured with PRBCs and uninfected RBCs in the absence and presence of TNF^low^. The sub-optimal dose was verified in separate TNF dose response studies; 5 pg/ml was 1/100th of the dose of TNF used as standard for optimal induction of ICAM-1 on HUVEC in our laboratory (0.5 ng/ml), and had no significant effect on ICAM-1 expression (data not shown). For the positive control a high dose of TNF (TNF^high^) was used (10 ng/ml) to stimulate the HUVEC, while medium alone served as a negative control. All parasite and EC cultures were regularly monitored for mycoplasma using the Takara PCR mycoplasma detection kit (Cambrex Biosciences).

### 2.3 PRBC-EC co-culture conditions

HUVEC grown to confluence in either 24-well plates or 25 cm^2^ flasks, for functional studies and microarray studies, respectively, were co-cultured with PRBCs and uninfected RBCs in the absence and presence of TNF^low^ at 37 °C. The RBC suspension was adjusted to 1% haematocrit. PRBC at the trophozoite stage (20–28 h post-invasion) were used in all co-culture studies; for the functional studies the parasites were synchronised using sorbitol, while for the microarray studies the trophozoites were additionally enriched using plasmagel flotation. In selected experiments, the parasites were retrieved after the 20-h, stained with giemsa and examined by light microscopy. There was no apparent rupture of RBC or change in the parasite stage over this period. For all functional studies the PRBCs were at a parasitaemia of 3% and co-cultured with HUVEC for 20 h. For microarray studies, the PRBCs were enriched on Plasmagel to parasitaemia ranging between 50% and 60% and co-cultured for 6 h. The uninfected RBCs were from the same batches used for parasite culture and were maintained under the same conditions as PRBCs, in separate flasks.

### 2.4 RNA expression: microarray analysis

Following incubation, the supernatant was aspirated, HUVEC was washed with cold RPMI-1640 and then with 0.02 M EDTA to remove the adherent RBCs and the cells harvested using Trizol (Invitrogen, UK). RNA integrity was evaluated by electrophoresis on a 1% agarose gel and by spectrophotometry using the absorbance ratio at 260/280 nm.

Five micrograms of each complementary biotin-labelled RNA was hybridised on the Affymetrix GeneChip, Human Genome version 2, HGU133plus2.0 (Santa Clara, CA), according to the manufacturer’s instructions. The signal intensity for each feature on the array was determined using the 70th percentile method provided by GCOS software (Affymetrix). Hybridisations were performed for four replicates of each of the seven conditions (control, RBC, PRBC, RBC + TNF^low^, PRBC + TNF^low^, TNF^low^ and TNF^high^).

Data analysis was performed using Bioconductor (Gentleman et al., 2004). The microarrays were pre-processed using a Robust Multiarray Averaging program (Irizarry et al., 2003). The expression values were calculated using the R statistical computing environment (http://www.r-project.org/) with the “affy” package (Gautier et al., 2004). The differential expression was assessed and variability estimated by fitting a linear model to the data that fully models the systematic part of each gene, using the “limma” package. From the “limma” output, log-fold changes from one condition compared with another, *P*-values corrected for a 5% false discovery rate (Hochberg and Benjamini, 1990), together with *B*-statistic (log-odds that a gene is differentially expressed) can be obtained and used to determine up- and down-regulation of genes. Due to the complexity of the microarray design (i.e. four replicates, seven conditions); for non-hierarchical clustering, the 47,000 transcripts present on the human chip were filtered down to 8105, based on their moderated *F*-statistic at *P*-value <0.001. These were then clustered by similarity. The unsupervised clustering program enabled us to discriminate biological clusters (ArrayMiner, OptimalDesign). For each cluster Gene Ontology (GO; http://www.geneontology.org) assignments were extracted using the GOstats package (http://bioconductor.org/packages/1.9/bioc/html/GOstats.html). The gene list of each cluster was used to identify all the unique GO terms in order to determine whether genes that comprise a given cluster have a common function, process or location in the cell. For each GO term, GOstats analysed whether that term was being significantly over-represented in the data, using a *P*-value cut-off of *P* ⩽ 0.05.

### 2.5 ICAM-1 protein expression

HUVEC grown to confluence in 24-well plates were co-cultured with PRBC and uninfected RBC at a haematocrit of 1% and a parasitaemia of 3% and suspended in M199 supplemented with 1% FCS in the absence and presence of 5 pg/ml TNF (TNF^low^) for 20 h at 37 °C. Following the incubation period, the supernatant was removed, centrifuged at 300*g* for 3 min to remove any RBCs and stored at −80 °C.

The HUVECs were washed once with cold RPMI-1640 and then with 0.02 M EDTA to remove the adherent RBCs and subsequently harvested by trypsinisation for analysis by flow cytometry. FACS ICAM-1 expression on HUVEC was determined by staining the cells using a fluorescein isothiocyanate (FITC)-conjugated mouse anti-human ICAM-1 antibody (MCA1615F; Serotec) using standard staining protocols and the cells fixed in 2% paraformaldehyde and analysed by flow cytometry. ICAM-1 expression was expressed as geometric mean of the fluorescence intensity.

### 2.6 IL-8 and TNF receptor expression

The supernatants stored from the co-culture studies were analysed using a standard sandwich ELISA kit (IDS), using a horse-radish peroxidise based colorimetric detection system, to quantify IL8 released from ECs. IL8 production was expressed as a concentration in pg per ml.

Similarly, soluble TNFR I, sTNFR I (p55) and soluble TNFR II, sTNFR II (p75), were detected using sTNFR I (KAC1761) and sTNFR II (KAC1771) ELISA kits (Biosource). TNFR level was expressed as TNFR concentration in ng per ml.

In order to understand the kinetics of TNFR expression on the surface of ECs in response to co-culture with PRBCs, the ECs were co-cultured with PRBCs and uninfected RBCs for 0.5, 1, 2 and 3 h. Following the incubation period, HUVECs were harvested and dual stained for surface TNFRs with monoclonal anti-human RII-FITC (FAB226F) and monoclonal anti-human RI-PE (FAB226F) antibodies (R&D Systems Europe). The receptor expression was expressed as the geometric mean of the fluorescence intensity.

### 2.7 Trypsin digestion of RBC

The ability of PRBCs to induce changes in surface ICAM-1 levels following trypsinisation was determined. PRBCs and uninfected RBCs were washed twice with PBS and incubated with 0.1 mg/ml trypsin in PBS for 15 min at room temperature with gentle mixing (modified from Chaiyaroj et al., 1994). After incubation the trypsin was inhibited with FCS at a final concentration of 10%. Conditions for trypsinisation were optimised to prevent cell lysis and the cells were stained with giemsa before and after trypsinisation to confirm RBC integrity was maintained. The RBCs were washed three times in PBS and resuspended in M199 supplemented with 1% FCS and co-cultured with HUVEC, as described.

We also analysed adhesion of PRBCs to microslides coated with 50 μg/ml Fc-tagged recombinant ICAM-1, ICAM-1-Fc (Gray et al., 2003), under laminar flow conditions at shear stress of 0.05 Pa, before and after trypsinisation, in order to evaluate PfEMP-1 mediated cytoadhesion.

### 2.8 Transwell experiments

Confluent HUVECs were co-cultured with PRBCs separated using a 0.4 μm transwell filter (Falcon), which prevents contact between HUVECs and RBCs but allows soluble factors to diffuse through. This set-up allowed us to determine whether contact between RBCs and ECs was necessary for induction of ICAM-1 expression on the ECs.

## 3 Results

### 3.1 Up-regulation of ICAM-1 expression following co-culture with infected and uninfected RBC

**Fig. 1:**
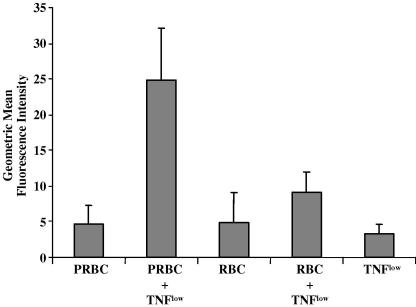
Up-regulation of intercellular adhesion molecule-1 expression on the surface of human umbilical vein endothelial cells co-cultured with *Plasmodium falciparum* srain ItG-infected and uninfected red blood cells (PRBC and RBC, respectively) in the absence and presence of 5 pg/ml TNF for 20 h. Data are given after subtraction of the control value and expressed as the mean of geometric mean of fluorescence ± SEM from 12 experiments. Statistical analysis of significance was performed using a paired *t*-test.

ICAM-1 is expressed constitutively at low levels on the surface of HUVEC in culture. Neither PRBCs nor uninfected RBCs was capable of inducing appreciable ICAM-1 expression over 20 h (Fig. 1). However, exposure to both PRBCs and TNF^low^ simultaneously, resulted in significant up-regulation of ICAM-1 expression (*P* < 0.05). TNF^low^ alone was incapable of inducing ICAM-1 expression (Fig. 1).

### 3.2 Up-regulation of IL-8 release following co-culture with infected and uninfected RBC

**Fig. 2:**
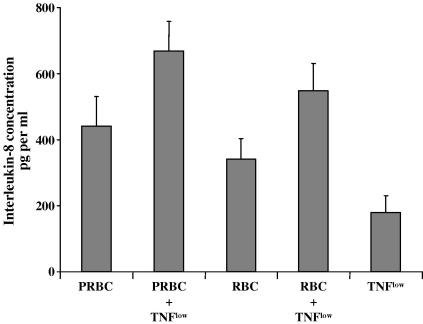
Up-regulation of IL-8 release from human umbilical vein endothelial cells co-culture with *Plasmodium falciparim* strain ItG-infected and uninfected red blood cells (PRBC and RBC, respectively) in the absence and presence of 5 pg/ml TNF for 20 h. Data are given after subtraction of the control value and expressed as the mean of geometric mean of fluorescence ± SEM from 10 experiments. Statistical analysis of significance was performed using a paired *t*-test.

Following incubation with either PRBCs or uninfected RBCs for 20 h, IL8 levels in the supernatant were increased over basal levels (*P* < 0.01) (Fig. 2). When the HUVECs were concurrently exposed to PRBCs and TNF^low^, IL8 release was further amplified over basal levels (*P* < 0.0002). Uninfected RBCs in combination with TNF^low^ induced similar levels of IL8 release (*P* < 0.001) (Fig. 2).

### 3.3 Up-regulation of soluble TNF receptor release and down-regulation of surface expression following co-culture with infected and uninfected RBC

**Fig. 3:**
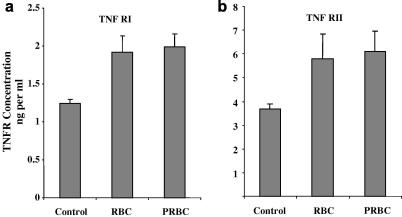
Up-regulation of soluble TNF receptors, TNFR I (a) and TNFR II (b), released into the supernatant from human umbilical vein endothelial cells, after co-culture with *Plasmodium falciparum* strain ItG-infected and uninfected red blood cells (PRBC and RBC, respectively) for 20 h, determined by ELISA. Expressed as mean concentration ± SEM, from four separate experiments.

Soluble forms of both TNFR I (p55) and TNFR II (p75) were significantly increased by 35% (*P* < 0.05) following treatment with either PRBCs or uninfected RBCs for 20 h. Thus, the phenomenon of soluble receptor release from the surface of ECs, when co-cultured with RBCs, appeared to be independent of the parasite (Fig. 3a and b).

**Fig. 4:**
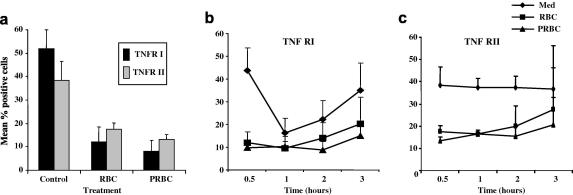
Analysis of the dynamics of the surface TNF receptor modulation in endothelial cells after co-culture with *Plasmodium falciparum* strain ItG-infected and uninfected red blood cells (PRBC and RBC, respectively). (a) Down-regulation of surface expression of TNF receptors I and II, over 30 min. (b) Changes in expression of TNFR I and (c) TNFR II monitored over 3 h; with PBRC and RBC. Expressed as mean percentage positive cells ± SEM, from four separate experiments.

Surface levels of the TNFRs were monitored over 3 h to understand the dynamics of TNFR expression. Both TNFR I and TNFR II were significantly reduced in response to both PRBCs and uninfected RBCs by 70% (*P* < 0.05) and 50% (*P* < 0.01), respectively, within 30 min (Fig. 4a). Interestingly, the levels of the TNFR I, but not TNFR II, fell over time under basal conditions, albeit at a slower rate than co-culture with RBCs. This could be a result of serum depletion when complete medium was replaced with M199/1% FCS for the duration of the co-culture experiment. In the case of both receptors, the observed down-regulation in response to RBCs was significantly greater than that seen under basal conditions.

### 3.4 Loss of ICAM-1-mediated adhesion phenotype following trypsinisation of PRBC

**Fig. 5:**
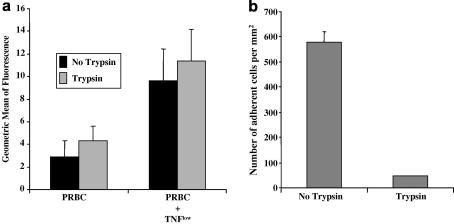
Investigation of the ability of trypsin to modulate the effects of *Plasmodium falciaprum* strain ItG-infected red blood cells (PRBC). (a) Up-regulation of intercellular adhesion molecule-1 (ICAM-1) on human umbilical vein endothelial cells induced by trypsin-treated PRBCs, in the presence of 5 pg/ml TNF. Expressed as mean of geometric mean of fluorescence ± SEM from four experiments. (b) Loss of intercellular adhesion molecule-1 (ICAM-1)-mediated adhesion of PRBC to immobilised ICAM-1-Fc under laminar flow conditions following trypsin-treatment. Expressed as mean number of adherent cells per mm^2^ ± SEM from three experiments.

Following trypsinisation, PRBCs did not lose the ability to up-regulate ICAM-1 expression on co-culture with ECs (Fig. 5a). As before, both PRBCs and uninfected RBCs induced an increase in surface levels of ICAM-1 in the presence of TNF^low^. ICAM-1 mediated adhesion under laminar flow, however, was abolished following trypsin treatment of PRBCs (Fig. 5b). In additional experiments, when the PRBCs were separated from the ECs in 24-well plates using 0.4 μm transwell inserts, there was no stimulation of ICAM-1 expression (data not shown). Thus, the EC activation observed in our system was critically dependent on cell–cell contact, although it did not involve specific PfEMP-1-mediated cytoadherence.

### 3.5 Modulation of the endothelial cell transcriptome following co-culture with infected and uninfected RBC

**Fig. 6:**
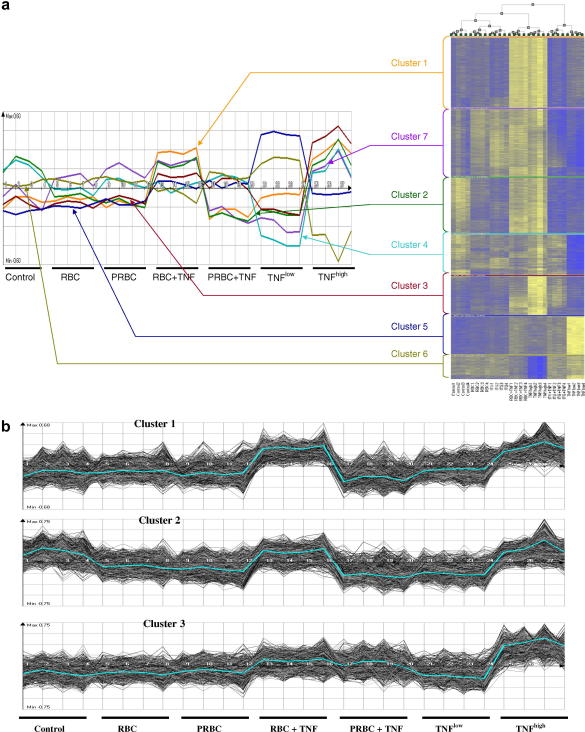
Analysis of microarray chips showing heatmap of gene expression and cluster analysis. (a) Modulation of the endothelial cell transcriptome following co-culture with *Plasmodium falciparum* strain ItG-infected and uninfected red blood cells (PRBC and RBC, respectively) in the absence and presence of 5 pg/ml TNF for 6 h, analysed using Affymetrix human genome chip and ArrayMiner software, showing seven non-hierarchical clusters with a total of 8105 genes, having a *P* value less than 0.001. (b) Three out of the seven clusters showing differential significant changes in gene expression. The data were taken from four separate experiments.

Using ArrayMiner software (OptimalDesign), seven non-hierarchical clusters were identified (Fig. 6a). As illustrated on the resulting heatmap, there is minimal change in the gene expression profiles with either PRBCs or RBCs compared with controls (Fig. 6b). However, when HUVECs were exposed to either PRBCs or RBCs in combination with TNF^low^, differential expression was visible in three out of the seven clusters, compared with control or TNF^low^ alone (Fig. 6a and b).

A comprehensive analysis of the microarray data was performed and has been submitted to Array Express (Accession No. E-SGRP-3). Our analysis focuses on genes that were differentially and significantly expressed at *P* < 0.001, compared with control levels. We have concentrated on over-represented gene ontologies and cited genes that best exemplify these GO terms.

In cluster 3, both PRBCs and uninfected RBCs, in combination with TNF^low^, produced over-expression (Fig. 6a and b). TNF^low^ alone, however, produced a significant under-expression. Genes in this cluster included the inflammatory protease, caspase 1, the acute response cytokine, IL8, inflammatory cytokines IFN-γ and lymphotoxin β, adhesion molecules ICAM-1, E-selectin and chemokines, CXCL2, CXCL3, CX3C1 and CXCL6.

In clusters 1 and 2, opposing effects were induced when HUVECs were exposed to either PRBCs or RBCs in combination with TNF^low^. Over-expression of genes was observed in response to uninfected RBCs and TNF^low^ and conversely, PRBCs and TNF^low^ resulted in under-expression of genes in these clusters (Fig. 6a and b).

In cluster 1, TNF^low^ on its own had no effect compared with controls, while in cluster 2 TNF^low^ alone produced a small reduction in gene expression (Fig. 6b). Genes in cluster 1 included the protease calpain 13, toll-like receptor-6 and the junction protein desmocollin 2, while genes in cluster 2 included the junction protein decorin, MHC class II, transferrin, endothelin 3 and endothelin receptor type A. While it is difficult to extrapolate roles for these genes from this in vitro analysis of the transcriptome, we can make some speculations based on recent studies. For instance, transferrin induction may be a mechanism for iron homeostasis to prevent accumulation of iron in endothelial cells which can lead to oxidative damage of the cells (Nanami et al., 2005). Endothelin is a known vasoconstrictor, in vivo; down-regulation of endothelin and its receptor may be a mechanism for maintaining vascular tone and integrity during malaria infection (Basilico et al., 2004). Down-regulation of MHC class II may represent suppression of the host immune response by the parasite.

**Fig. 7:**
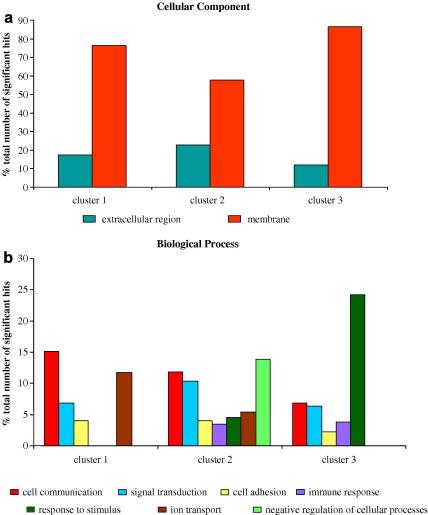
Evaluation of proportions of gene ontology (GO) terms over-represented, for all three clusters,with respect to (a) cellular component and (b) biological process. The graphs represent proportions of GO terms that have ⩾2% of the total number of significant hits at *P* < 0.05. GO terms that have <2% of the total number of hits have been excluded from these graphs. Fig. S1 (supplementary data) shows a full analysis of all GO terms with ⩾2% significant hits and Supplementary Tables 1, 2 and 3 show a detailed list of all GO terms with <2% significant hits.

The genes in each cluster were further analysed and categorised by GO) terms using the GOstats package of R/Bioconductor to understand their basic biological functions. The over-represented GO terms, defining genes in each cluster, in terms of biological process, cellular component and molecular function, showing ⩾2% of the total number of genes being annotated with that term, were evaluated. Fig. 7 illustrates distribution of the over-represented gene ontologies with respect to biological process and cellular component, in clusters 1 and 2, which reflect inflammatory and protective functions in the endothelial cells. The full analysis for all three clusters is detailed in Supplementary Fig. S1(see on-line supplementary data). Whilst some GO terms fell below the 2% cut-off, it is important to note that they were nonetheless significant and have been tabulated for further information (Supplementary Tables 1, 2 and 3; see on-line supplementary data). A wide range of GO terms were over-represented in each cluster within categories of biological process, cellular component and molecular function, with a number of common and parallel themes, including cell communication, signal transduction, cell adhesion, organismal physiological process, ion transport, response to external stimuli and immune response. Interestingly, the genes in each of these clusters were primarily cell membrane-associated, or related to the extracellular region and the extracellular matrix. The molecular functions of the genes in each cluster were wide ranging, although receptor activity, signal transducer activity, ion channel and protein binding activities were parallel themes in all three clusters.

The fold changes for the 8105 genes were calculated for each of the conditions for visualizing the changes in expression, compared with control levels. Expression of ICAM-1 and IL8 RNA was up-regulated, reaching 3.7-fold and 7-fold, respectively, over control levels (*P* = 0.001), when HUVECs were incubated with a combination of PRBCs and TNF^low^. Interestingly, ICAM-1 transcription reached similar levels in the presence of a combination of uninfected RBCs and TNF^low^. Table 1 illustrates the changes in ICAM-1 and IL8 expression under the different co-culture conditions.

Table 2 shows the changes in expression of selected genes which illustrate specific biological processes of ECs that may have a role in mediating either pathology or protection, and their respective fold changes in the presence of PRBCs, uninfected RBCs and TNF^low^ compared with controls. Although the magnitudes of change are relatively small, they are highly reproducible and biologically significant at *P* < 0.001, over four separate replicates.

The chemokines, CXCL2 (Gro-β), CXCL3 (Gro-γ), CXCL6 and E-selectin were all over-expressed in the presence of PRBCs and TNF^low^, with variable increases in expression of CXCL2 and CXCL6 in the presence of uninfected RBCs and TNF^low^ (Table 2). Gro-β has been shown to have a role in the adhesion of monocytes to ECs (Schwartz et al., 1994) while CXCL6 has similar properties to IL8 and can be co-induced with IL8 (Gijsbers et al., 2005). E-selectin was also over-expressed in response to PRBCs, uninfected RBCs and TNF^low^, reaching up to 7-fold over controls with PRBCs and TNF^low^.

Molecules involved in cell adhesion such as ICAM-1 and CX3C1 were both over-expressed in PRBCs, uninfected RBCs, and TNF^low^, with a greater effect in PRBCs (Table 2). CX3C1 (fractalkine) has recently been implicated in mediating adhesion of PRBCs to ECs (Hatabu et al., 2003). Other adhesion receptors such as desmocollin 2 and integrin β1 were both under-expressed with PRBCs and TNF, and over-expressed with uninfected RBCs and TNF (Table 2).

A number of inflammatory genes such as MHC class I, superoxide dismutase 2 (SOD 2) and lymphotoxin-β were all over-expressed in the presence of PRBCs and TNF^low^ (Table 2). Whilst both MHC class I and SOD 2 were also over-expressed with uninfected RBCs and TNF^low^, lymphotoxin β was under-expressed.

Table 2 illustrates differential expression of molecules such as TNFR-associated factor 1 (TRAF 1), Fas associated factor 1 (FAF-1), prostaglandin receptor 3 and TGF-β3, which all have a role in signal transduction.

A number of cell apoptosis-related genes were differentially regulated in our co-culture system (Table 3). Both caspase 3 and caspase 9, which are believed to be inducers of apoptosis (Ho and Hawkins, 2005), were under-expressed in the presence of PRBCs and TNF^low^. In addition, caspase 3 expression was markedly increased in the presence of uninfected RBCs and TNF. Calpains, which are inducers of cell death via a caspase-independent pathway (Utz and Anderson, 2000; Perrin and Huttenlocher, 2002), were also regulated in our system. We looked at the expression of two calpains, 9 and 13. Calpain 9 was under-expressed and there was a small increase in calpain 13 expression in the presence of PRBCs and TNF^low^ (Fig. 8). Thus, our data does not suggest induction of apoptosis; in fact, there appears to be a trend towards protection from apoptosis and this was supported by our inability to detect any positive staining with annexin V and the absence of any morphological changes in the ECs after co-culture.

## 4 Discussion

Sequestration of PRBCs at vascular sites is a critical event in the pathogenesis of severe malaria. Here we have investigated the effect of prolonged exposure to PRBCs on the endothelium. We have demonstrated a phenomenon whereby ECs are differentially modulated when co-cultured with PRBCs in the presence of TNF^low^, which could occur at microvascular sites early in infection, for instance during schizogony. Unlike previously published work, in our system there was no apparent induction of ICAM-1 or modulation of RNA transcription by PRBCs alone. Significant de novo ICAM-1 induction and differential regulation of gene transcription were dependent on the presence of low levels of TNF. PRBCs alone did modulate certain EC functions such as IL8 release and TNFR shedding from the cell surface, but IL8 release was further enhanced in the presence of TNF^low^.

Up-regulation of ICAM-1 was maintained following removal of surface expressed PfEMP-1 by trypsin digestion, but was abolished when cell–cell contact was prevented. This demonstrated that while cell-to-cell contact was crucial for modulating EC function, there was no requirement for adhesion via specific receptors in this model. Despite the differences in the reported effects of PRBCs on ECs, the unifying theme in all the co-culture studies is this critical requirement for close apposition of the two cell types in the co-culture system, in the induction of EC activation. The transwell system, whilst preventing cell-to-cell contact, allowed the movement of parasite-derived soluble factors. Therefore we could exclude the role of soluble parasite-derived factors in EC activation. In all cases, when the cell–cell contact was compromised using a filter, EC activation was abolished (Randolph and Furie, 1996; Pino et al., 2003; Viebig et al., 2005). Thus the variable observations may reflect differences in the respective co-culture conditions, for instance, studies by Viebig et al. (2005) were performed in complete HUVEC culture medium and studies by Pino et al. (2003) were performed in parasite culture media, while our studies were performed in a serum-depleted basal HUVEC medium in order to enhance the signal window for the EC measurements, whilst maintaining EC integrity. It is clear from our work and that of others, that care will need to be taken in interpreting the results of model co-culture systems and that further work will be required to develop ex vivo models of severe disease. It is possible that no single model will be able to reflect the variable pathology represented in malaria infection. Our study is unique in the observation that concomitant presence of low level TNF is necessary for PRBCs to modulate the endothelium, namely HUVECs. Indeed, a recent study also suggested that PRBCs alone do not have the ability to induce ICAM-1 in HUVECs, and that this may be a phenomenon specific to brain-derived ECs (Tripathi et al., 2006).

Using HUVEC as an in vitro model for studying PRBC-EC interactions in vivo, we have demonstrated that close apposition or cell–cell contact of the PRBCs with ECs is a critical factor in mediating this activation. Whilst uninfected RBCs also had an effect on ECs in the presence of low level TNF, it is noteworthy that in vivo, apposition of uninfected RBCs on ECs only occurs secondary to cytoadhesion or as a result of vessel occlusion. The ability of normal RBCs to stick to and modulate ECs in vitro, however, is not a novel observation. Indeed, normal RBCs can bind to ECs mediated by plasma factors such as fibrinogen and fibronectin (Wautier et al., 1983) and the binding can induce low-grade ICAM-1 expression in vitro (Brown et al., 2001b). RBCs express a wide range of molecules on their surface which have been implicated in mediating adhesion to endothelial cells under different conditions. These include: (i) VLA-4 (alpha4beta1) which can bind to endothelial VCAM-1 (Walmet et al., 2003); (ii) the blood group Lutheran molecule (LU) over-expressed on sRBCs can bind to laminin present on cells or in the intercellular space (Eyler and Telen, 2006); (iii) advanced glycation end products (AGEs) present on RBCs bind to their receptor (RAGE) on endothelium (Wautier and Schmidt, 2004), activating endothelial cells; and (iv) a molecule related to blood group Rhesus, ICAM-4 (Hermand et al., 2003) binds to integrins present on leukocytes (CD11–CD18) and on platelets (alpha2beta4) offering a surface which can be involved in thrombosis (reviewed by Wautier and Wautier, 2004). Thus, although PfEMP-1 does not play a role in the induction of ICAM-1 expression seen by us, it would normally be required to bring the PRBCs and ECs together in vivo.

Contrary to our hypothesis that up-regulation of TNFRs in response to PRBCs contributed to the increased sensitivity to TNF, unexpectedly, we observed significant down-regulation of surface TNFR expression and increased receptor shedding. It is possible that the enhancement of the effects of RBCs is at the level of intracellular signal transduction. Rapid TNFR shedding by cleavage of the extracellular region of the receptors as observed in our studies is a common response to various stimuli (Aderka et al., 1998) and may be a mechanism for down-modulation of both TNF receptors. This rapid release of TNFRs in response to an insult to the endothelium or physical cell contact with the endothelium is believed to be a non-specific pro-inflammatory response and up-regulation of soluble TNFR I has been correlated with parasitaemia in the serum of patients with acute *P. falciparum* infection (Wenisch et al., 1994). The TNFR shedding seen in response to PRBCs or uninfected PRBCs alone, may be part of a non-specific inflammatory response of ECs to prolonged cell–cell contact. Similarly, IL8 release in our system may also represent a non-specific inflammatory response to cell–cell contact with RBCs, as was demonstrated in a monocyte/EC co-culture model and was further shown to be independent of ICAM-1 mediated binding (Lukacs et al., 1995).

Similarly to our direct measurements of ICAM-1, there was no significant change in EC gene transcription in response to PRBCs or RBCs alone but in combination with TNF^low^, significant regulation of the EC transcriptome was induced. Analysis of the transcriptome revealed a wide diversity of gene ontologies representing genes that were significantly regulated, however, care must be taken in interpreting the data since there is a high degree of redundancy across GO terms, which was not accounted for in this analysis. IL8, for instance, has diverse biological roles including cell communication, cell adhesion, signal transduction and immune response. In spite of such deficiencies, the GO terms that were over-represented, including cell communication, cell adhesion, signal transduction, ion transport and immune response, fit well with our hypothesis that the infected RBC is able to modulate the host endothelial response.

From previous work, we had expected to see a pro-inflammatory response induced by PRBCs (cluster 3, Fig. 6b). Less predictable was the down-regulation of genes in the presence of PRBCs and TNF^low^ (clusters 1 and 2, Fig. 6b), which may represent a parasite-specific adaptive response that favours parasite survival and development by protecting the integrity of the host endothelium. Up-regulation of these genes under the same conditions may result in the induction of a pro-adhesive effect that can potentially feed back to reinforce pathological sequestration that is typically seen in the later stages of severe malaria. In addition, induction of an immune response, as in cluster 3, may confer a protective effect on the host. Thus, we propose that the balance between these converse effects of PRBCs and TNF on the endothelium may be the critical factor in determining the response by endothelium and the clinical outcome of a malaria infection in vivo.

The over-expression of genes in response to uninfected RBCs and TNF^low^ in two of the clusters, whilst unexpected, is a common phenomenon in static in vitro co-culture studies. For instance, normal RBCs can interact with ECs to induce the expression of a variety of pro-inflammatory molecules including ICAM-1 (Brown et al., 2001b), VCAM-1 (Brown et al., 2001b; Wautier et al., 2001) and activation of NADPH oxidase, which mediates the generation of reactive oxygen intermediates (Wautier et al., 2001). This is a potential mechanism to induce expression of genes to counteract the response to PRBCs and may reflect a partially protective host response during a malaria infection, balanced against inducing a pro-adhesive environment, particularly early in infection when systemic inflammation is unlikely to be a major contributor to pathogenesis.

Modulation of host cells has been demonstrated in other co-culture models, for instance tyrosine phosphorylation of scyncitiotrophoblasts in a model of placental malaria (Lucchi et al., 2006) and ecto-phosphorylation of CD36 following the initial PRBC attachment which is believed to further enhance CD36-mediated cytoadhesion (Yipp et al., 2003). While co-culture models give useful insights into mechanisms of action of PRBCs on host cells in malaria pathogenesis, care must be taken in the interpretation and extrapolation of this data to pathogenesis in vivo, since these mechanisms may not be reflected in the in vivo situation.

In summary, our co-culture model identifies a novel mechanism whereby PRBCs, whilst mobilizing a pro-inflammatory and a seemingly pro-adhesive effect on the host endothelium, have the additional ability to suppress gene expression pathways involved in signal transduction, apoptosis and immune response as part of a mechanism to support parasite survival through maintaining the overall integrity of the host EC. This fits well with histo-pathological observations in severe malaria where transient dysfunction of the blood–brain barrier is observed (Brown et al., 1999a) but the overall health of the endothelium seems to be maintained despite the presence of many adherent PRBCs (Medana and Turner, 2006). Our suggestion is that the host response to sequestration is complex and involves a balance between pro- and anti-inflammatory pathways, both mediated by apposition of the PRBC, and interacting with other mechanisms of host response to malaria infection variably throughout an infection. This balance is critical to the health of an EC and the resulting pathology associated with severe malaria.

## Figures and Tables

**Table 1:** Intercellular adhesion molecule-1 (ICAM-1) and IL8 RNA expression expressed as fold changes compared with control

	PRBC	PRBC+TNF^low^	RBC	RBC+TNF^low^	TNF^low^
ICAM-1	1.85	3.71	2.07	3.38	2.41
IL-8	4.23	7.14	2.12	2.97	2.38

Values greater than 1 denote over-expression of a gene and values less than 1 denote under-expression of a gene at *P* < 0.001. PRBC, *Plasmodium falciparum*-infected red blood cells.

**Table 2:** Expression of selected genes representing specific biological processes in endothelial cells, such as cell communication, cell adhesion, organismal physiological process and signal transduction

GO term	Genes	PRBC+TNF^low^	RBC+TNF^low^
Cell communication	E-selectin	6.89	3.07
	CXCL2	2.74	1.04
	CXCL3	2.28	0.71
	CXCL6	2.82	2.16
			
Cell adhesion	ICAM-1	3.71	3.38
	CX3C1	1.76	1.15
	Desmocollin 2	0.89	1.42
	Integrin β1	0.85	1.28
			
Organismal physiological process	MHC class I	1.40	1.48
	SOD2	1.49	1.16
	Lymphotoxin β	1.24	0.93
			
Signal transduction	TRAF-1	1.92	1.34
	TGF β3	1.34	1.53
	PGE R3	1.17	1.8
	FAF-1	0.53	0.99

The table illustrates fold changes in response to red blood cells (RBC) (infected and uninfected) and TNF, compared with control. Values greater than 1 denote over-expression of a gene and values less than 1 denote under-expression of a gene at *P* < 0.001. PRBC, *Plasmodium falciparum*-infected red blood cells.

**Table 3:** Changes in expression of selected protease genes with a potential role in apoptosis, in endothelial cells

	PRBC + TNF^low^	RBC + TNF^low^
Caspase 1	1.27	0.91
Caspase 3	1.02	1.31
Caspase 9	0.91	0.89
Calpain 9	0.81	1.05
Calpain 13	1.13	1.94

The table illustrates fold changes in response to re blood cells (RBC) (infected and uninfected) and TNF, compared with control. Values greater than 1 denote over-expression of a gene and values less than 1 denote under-expression of a gene at *P* < 0.001. PRBC, *Plasmodium falciparum*-infected red blood cells.
