# Supplementary material for: Altered phenotype and gene transcription in endothelial cells, induced by Plasmodium falciparum-infected red blood cells: Pathogenic or protective?
Source: Int J Parasitol. 2007 Jul;37(8-9):975–87. doi: 10.1016/j.ijpara.2007.02.006 (PMC1906861; doi:10.1016/j.ijpara.2007.02.006)
Supplement: Supplementary data [file mmc1.doc]

**Supplementary Table 1. Cluster 1: Gene ontology (GO) annotations that have < 2% of the total number of significant hits at *P* < 0.05.**

| Biological Process |  | Cellular Component |  | Molecular Function |  |
| --- | --- | --- | --- | --- | --- |
| GO Term | % | GO Term | % | GO Term | % |
| transmembrane receptor protein tyrosine kinase signaling pathway | 1.1363636 | basolateral plasma membrane | 0.3432494 | ligand-gated ion channel activity | 1.6336056 |
| enzyme linked receptor protein signaling pathway | 1.6528926 | voltage-gated sodium channel complex | 0.3432494 | GABA-A receptor activity | 0.5834306 |
| inorganic anion transport | 1.1363636 | postsynaptic membrane | 0.9153318 | voltage-gated ion channel activity | 1.8669778 |
| humoral immune response | 1.2396694 | dystrophin-associated glycoprotein complex | 0.3432494 | calcium channel activity | 0.9334889 |
| muscle contraction | 1.2396694 | membrane coat adaptor complex | 0.3432494 | GABA receptor activity | 0.5834306 |
| synaptic transmission | 1.7561983 | myofibril | 0.5720824 | extracellular ligand-gated ion channel activity | 0.9334889 |
| transmission of nerve impulse | 1.8595041 | protein phosphatase type 2A complex | 0.3432494 | L-amino acid transporter activity | 0.4667445 |
| regulation of organismal physiological process | 1.1363636 | basal lamina | 0.4576659 | cytokine activity | 1.9836639 |
| inactivation of MAPK activity | 0.3099174 | contractile fiber | 0.5720824 | ligase activity, forming carbon-nitrogen bonds | 1.6336056 |
| ossification | 0.5165289 | protein serine/threonine phosphatase complex | 0.4576659 | potassium channel activity | 1.2835473 |
| negative regulation of protein kinase activity | 0.5165289 | sarcomere | 0.4576659 | MHC class II receptor activity | 0.3500583 |
| calcium ion transport | 0.8264463 | synapse | 1.1441648 | metallopeptidase activity | 1.6336056 |
| calcium ion homeostasis | 0.7231405 |  |  | actin binding | 1.9836639 |
| metal ion homeostasis | 0.9297521 |  |  | Rho GTPase binding | 0.3500583 |
| complement activation | 0.5165289 |  |  | immunoglobulin binding | 0.3500583 |
| epidermal growth factor receptor signaling pathway | 0.4132231 |  |  | voltage-gated sodium channel activity | 0.3500583 |
| transforming growth factor beta receptor signaling pathway | 0.4132231 |  |  | phosphoric diester hydrolase activity | 0.8168028 |
| gamma-aminobutyric acid signaling pathway | 0.3099174 |  |  | ubiquitin-protein ligase activity | 1.1668611 |
| glutamate signaling pathway | 0.3099174 |  |  | carboxypeptidase activity | 0.5834306 |
| learning and/or memory | 0.3099174 |  |  | amine transporter activity | 0.8168028 |
| amino acid catabolism | 0.6198347 |  |  | protein phosphatase regulator activity | 0.5834306 |
| amine catabolism | 0.6198347 |  |  | hematopoietin/interferon-class (D200-domain) cytokine receptor activity | 0.7001167 |
| negative regulation of signal transduction | 0.6198347 |  |  | voltage-gated potassium channel activity | 0.9334889 |
| L-amino acid transport | 0.4132231 |  |  | extracellular matrix structural constituent | 0.9334889 |
| Biological Process |  | Cellular Component |  | Molecular Function |  |
| GO Term | % | GO Term | % | GO Term | % |
| amine transport | 0.7231405 |  |  | phosphatase regulator activity | 0.5834306 |
| antigen presentation, exogenous antigen | 0.3099174 |  |  | ionotropic glutamate receptor activity | 0.3500583 |
| antigen processing, exogenous antigen via MHC class II | 0.3099174 |  |  | glutamate-gated ion channel activity | 0.3500583 |
| di-, tri-valent inorganic cation homeostasis | 0.9297521 |  |  | protein phosphatase type 2A regulator activity | 0.3500583 |
| lymphocyte differentiation | 0.5165289 |  |  | cyclic nucleotide binding | 0.3500583 |
| T cell differentiation | 0.4132231 |  |  | anion transporter activity | 0.9334889 |
| biomineral formation | 0.5165289 |  |  |  |  |
| T cell activation | 0.6198347 |  |  |  |  |
| negative regulation of MAPK activity | 0.3099174 |  |  |  |  |
| nitrogen compound catabolism | 0.6198347 |  |  |  |  |
| lymphocyte activation | 0.9297521 |  |  |  |  |
| bone remodeling | 0.7231405 |  |  |  |  |
| tissue remodeling | 0.7231405 |  |  |  |  |
| positive regulation of lymphocyte proliferation | 0.3099174 |  |  |  |  |
| regulation of T cell activation | 0.4132231 |  |  |  |  |
| positive regulation of organismal physiological process | 0.6198347 |  |  |  |  |
| negative regulation of transferase activity | 0.5165289 |  |  |  |  |
| cell activation | 1.0330579 |  |  |  |  |
| muscle development | 1.0330579 |  |  |  |  |
| di-, tri-valent inorganic cation transport | 1.0330579 |  |  |  |  |
| immune cell activation | 1.0330579 |  |  |  |  |

**Supplementary Table 2. Cluster 2: Gene ontology (GO) annotations that have < 2% of the total number of significant hits at *P* < 0.05.**

| Biological Process |  | Cellular Component |  | Molecular Function |  |
| --- | --- | --- | --- | --- | --- |
| GO Term | % | GO Term | % | GO Term | % |
| cellular defense response | 0.944206 | neuron projection | 0.7246377 | 3',5'-cyclic-nucleotide phosphodiesterase activity | 1.2468828 |
| steroid metabolism | 0.944206 | axoneme | 0.7246377 | cyclic-nucleotide phosphodiesterase activity | 1.2468828 |
| cell-cell adhesion | 1.2017167 | cell projection | 1.9323671 | glutamate receptor activity | 1.4962594 |
| transmission of nerve impulse | 1.5450644 | collagen | 1.2077295 | ionotropic glutamate receptor activity | 0.9975062 |
| metal ion transport | 1.9742489 |  |  | glutamate-gated ion channel activity | 0.9975062 |
| C21-steroid hormone biosynthesis | 0.2575107 |  |  | endonuclease activity, active with either ribo- or deoxyribonucleic acids and producing 3'-phosphomonoesters | 0.9975062 |
| xenobiotic metabolism | 0.3433476 |  |  | pancreatic ribonuclease activity | 0.7481297 |
| phosphate transport | 0.6008584 |  |  | GTPase binding | 1.7456359 |
| iron ion transport | 0.2575107 |  |  | small GTPase binding | 1.4962594 |
| muscle contraction | 1.1158798 |  |  | phosphoric diester hydrolase activity | 1.7456359 |
| smooth muscle contraction | 0.3433476 |  |  | endoribonuclease activity, producing 3'-phosphomonoesters | 0.7481297 |
| regulation of mitosis | 0.4291845 |  |  | non-membrane spanning protein tyrosine kinase activity | 0.7481297 |
| heterophilic cell adhesion | 0.3433476 |  |  | growth factor binding | 1.4962594 |
| phospholipase C activation | 0.2575107 |  |  | di-, tri-valent inorganic cation transporter activity | 0.9975062 |
| synaptic transmission | 1.4592275 |  |  | carboxylesterase activity | 0.7481297 |
| sensory perception of sound | 0.6866953 |  |  | serine esterase activity | 0.7481297 |
| C21-steroid hormone metabolism | 0.2575107 |  |  | endoribonuclease activity | 0.9975062 |
| hexose transport | 0.2575107 |  |  |  |  |
| aspartate family amino acid metabolism | 0.2575107 |  |  |  |  |
| response to xenobiotic stimulus | 0.4291845 |  |  |  |  |
| monosaccharide transport | 0.2575107 |  |  |  |  |
| glucose transport | 0.2575107 |  |  |  |  |
| regulation of cyclase activity | 0.3433476 |  |  |  |  |
| hormone metabolism | 0.6008584 |  |  |  |  |
| hormone biosynthesis | 0.3433476 |  |  |  |  |
| negative regulation of apoptosis | 0.8583691 |  |  |  |  |
| regulation of adenylate cyclase activity | 0.3433476 |  |  |  |  |
| sensory perception of mechanical stimulus | 0.6866953 |  |  |  |  |
| negative regulation of transport | 0.2575107 |  |  |  |  |
| regulation of lyase activity | 0.3433476 |  |  |  |  |
| central nervous system development | 0.8583691 |  |  |  |  |
| di-, tri-valent inorganic cation transport | 0.8583691 |  |  |  |  |

**Supplementary Table 3. Cluster 3: Gene ontology (GO) annotations that have < 2% of the total number of significant hits at *P* < 0.05.**

| Biological Process |  | Cellular Component |  | Molecular Function |  |
| --- | --- | --- | --- | --- | --- |
| GO Term | % | GO Term | % | GO Term | % |
| nervous system development | 1.3182674 | MHC protein complex | 0.4219409 | phospholipid-translocating ATPase activity | 1.0309278 |
| sensory perception | 1.3182674 | MHC class I protein complex | 0.4219409 | aminophospholipid transporter activity | 1.0309278 |
| behavior | 0.8788449 | collagen | 0.5625879 | ATPase activity | 1.5463918 |
| locomotory behavior | 0.8160703 |  |  | di-, tri-valent inorganic cation transporter activity | 1.2886598 |
| response to abiotic stimulus | 1.4438167 |  |  | phospholipid transporter activity | 1.0309278 |
| morphogenesis | 1.6321406 |  |  | calcium ion transporter activity | 0.7731959 |
| organ morphogenesis | 0.7532957 |  |  | Rab GTPase binding | 0.7731959 |
| cell differentiation | 1.569366 |  |  | porin activity | 0.7731959 |
| taxis | 0.8160703 |  |  | oxidoreductase activity | 1.0309278 |
| organ development | 1.8204645 |  |  | voltage-gated chloride channel activity | 0.7731959 |
| system development | 1.3182674 |  |  | antigen binding | 1.0309278 |
| response to wounding | 1.6949153 |  |  | protein tyrosine/serine/threonine phosphatase activity | 1.0309278 |
| cell proliferation | 1.6321406 |  |  | protein tyrosine phosphatase activity | 1.5463918 |
| cell death | 1.569366 |  |  | carbohydrate kinase activity | 0.7731959 |
| death | 1.569366 |  |  | oxidoreductase activity | 1.8041237 |
| response to chemical stimulus | 1.2554928 |  |  | unspecific monooxygenase activity | 0.7731959 |
| inflammatory response | 1.0671689 |  |  | subtilase activity | 0.5154639 |
| regulation of apoptosis | 1.0671689 |  |  | calcium-activated potassium channel activity | 0.5154639 |
| regulation of programmed cell death | 1.0671689 |  |  | antiporter activity | 1.0309278 |
| regulation of cell proliferation | 1.0043942 |  |  |  |  |
| chemotaxis | 0.8160703 |  |  |  |  |
| cell-cell adhesion | 0.8160703 |  |  |  |  |
| cellular morphogenesis during differentiation | 0.4394225 |  |  |  |  |
| angiogenesis | 0.4394225 |  |  |  |  |
| blood vessel development | 0.4394225 |  |  |  |  |
| cell activation | 0.5021971 |  |  |  |  |
| vasculature development | 0.4394225 |  |  |  |  |
| phosphate transport | 0.3766478 |  |  |  |  |
| anion transport | 0.6277464 |  |  |  |  |
| neurotransmitter transport | 0.3138732 |  |  |  |  |
| lipid transport | 0.3766478 |  |  |  |  |
| humoral immune response | 0.5649718 |  |  |  |  |
| cellular defense response | 0.5021971 |  |  |  |  |
| axonogenesis | 0.3766478 |  |  |  |  |
| muscle development | 0.4394225 |  |  |  |  |
| striated muscle development | 0.3138732 |  |  |  |  |
| circulation | 0.5021971 |  |  |  |  |
| blood pressure regulation | 0.2510986 |  |  |  |  |
| response to virus | 0.3766478 |  |  |  |  |
| response to bacteria | 0.3766478 |  |  |  |  |
| inorganic anion transport | 0.5649718 |  |  |  |  |
| calcium-independent cell-cell adhesion | 0.1883239 |  |  |  |  |
| antigen presentation | 0.2510986 |  |  |  |  |
| antigen presentation, endogenous antigen | 0.2510986 |  |  |  |  |
| antigen processing, endogenous antigen via MHC class I | 0.4394225 |  |  |  |  |
| regulation of cell adhesion | 0.2510986 |  |  |  |  |
| neuron differentiation | 0.5649718 |  |  |  |  |
| extracellular matrix organization and biogenesis | 0.2510986 |  |  |  |  |
| antigen processing | 0.4394225 |  |  |  |  |
| neurite morphogenesis | 0.4394225 |  |  |  |  |
| T cell proliferation | 0.1883239 |  |  |  |  |
| positive regulation of T cell proliferation | 0.1883239 |  |  |  |  |
| regulation of T cell proliferation | 0.1883239 |  |  |  |  |
| lipoprotein metabolism | 0.2510986 |  |  |  |  |
| extracellular structure organization and biogenesis | 0.2510986 |  |  |  |  |
| immune cell activation | 0.5021971 |  |  |  |  |
| lymphocyte activation | 0.3766478 |  |  |  |  |
| lymphocyte proliferation | 0.2510986 |  |  |  |  |
| negative regulation of nucleocytoplasmic transport | 0.1255493 |  |  |  |  |
| cell development | 0.6277464 |  |  |  |  |
| blood vessel morphogenesis | 0.4394225 |  |  |  |  |
| skeletal muscle development | 0.1883239 |  |  |  |  |
| neuron development | 0.5649718 |  |  |  |  |
| neuron morphogenesis during differentiation | 0.4394225 |  |  |  |  |
| neurogenesis | 0.5649718 |  |  |  |  |
| skeletal muscle fiber development | 0.1883239 |  |  |  |  |
| muscle fiber development | 0.1883239 |  |  |  |  |
| regulation of lymphocyte proliferation | 0.2510986 |  |  |  |  |
| positive regulation of lymphocyte proliferation | 0.1883239 |  |  |  |  |
| regulation of immune response | 0.3766478 |  |  |  |  |
| regulation of cell activation | 0.2510986 |  |  |  |  |
| positive regulation of cell activation | 0.1883239 |  |  |  |  |
| positive regulation of T cell activation | 0.1883239 |  |  |  |  |
| negative regulation of protein transport | 0.1255493 |  |  |  |  |
| regulation of lymphocyte activation | 0.2510986 |  |  |  |  |
| positive regulation of lymphocyte activation | 0.1883239 |  |  |  |  |

Supplementary Fig. S1: The pie charts show gene ontology (GO) annotations in terms of biological process, cellular component and molecular function of all three clusters for all genes that have ≥ 2% of the total number of significant hits at *P* < 0.05. GO terms in red represent commonality between the three clusters.
